# Cost of cardiovascular disease events in patients with and without type 2 diabetes and factors influencing cost: a retrospective cohort study

**DOI:** 10.1186/s12889-024-19475-w

**Published:** 2024-07-26

**Authors:** Sharifa Ezat Wan Puteh, Noor ‘Adilah Kamarudin, Zanariah Hussein, Noorlita Adam, Mohd Ridzwan Shahari

**Affiliations:** 1 Department of Public Health Medicine, Faculty of Medicine, Universiti Kebangsaan Malaysia, Jalan Yaacob Latif, Kuala Lumpur, 56000 Cheras Wilayah Persekutuan Malaysia; 2 Department of Medical, Hospital Putrajaya, Wilayah Persekutuan Putrajaya, Putrajaya, Malaysia; 3 Department of Internal Medicine, Hospital Tuanku Ja’afar, Seremban, Negeri Sembilan Malaysia; 4 Medical Division, Ministry of Health, Putrajaya, Malaysia

**Keywords:** Cardiovascular disease, Cardiovascular disease event, Type 2 diabetes mellitus, Hospitalisation, Treatment cost, Casemix, Diagnosis Related Group

## Abstract

**Background:**

Cardiovascular disease (CVD) and type 2 diabetes mellitus (T2DM) are non-communicable diseases that impose a significant economic burden on healthcare systems, particularly in low- and middle-income countries. The purpose of this study was to evaluate the hospital treatment cost for cardiovascular disease events (CVDEs) in patients with and without diabetes and identify factors influencing cost.

**Method:**

We conducted a retrospective, cross-sectional study using administrative data from three public tertiary hospitals in Malaysia. Data for hospital admissions between 1 March 2019 and 1 March 2020 with International Classification of Diseases 10th Revision (ICD-10) codes for acute myocardial infarction (MI), ischaemic heart disease (IHD), hypertensive heart disease, stroke, heart failure, cardiomyopathy, and peripheral vascular disease (PVD) were retrieved from the Malaysian Disease Related Group (Malaysian DRG) Casemix System. Patients were stratified by T2DM status for analyses. Multivariate logistic regression was used to identify factors influencing treatment costs.

**Results:**

Of the 1,183 patients in our study cohort, approximately 60.4% had T2DM. The most common CVDE was acute MI (25.6%), followed by IHD (25.3%), hypertensive heart disease (18.9%), stroke (12.9%), heart failure (9.4%), cardiomyopathy (5.7%) and PVD (2.1%). Nearly two-thirds (62.4%) of the patients had at least one cardiovascular risk factor, with hypertension being the most prevalent (60.4%). The treatment cost for all CVDEs was RM 4.8 million and RM 3.7 million in the T2DM and non-T2DM group, respectively. IHD incurred the largest cost in both groups, constituting 30.0% and 50.0% of the total CVDE treatment cost for patients with and without T2DM, respectively. Predictors of high treatment cost included male gender, non-minority ethnicity, IHD diagnosis and moderate-to-high severity level.

**Conclusion:**

This study provides real-world cost estimates for CVDE hospitalisation and quantifies the combined burden of two major non-communicable disease categories at the public health provider level. Our results confirm that CVDs are associated with substantial health utilisation in both T2DM and non-T2DM patients.

**Supplementary Information:**

The online version contains supplementary material available at 10.1186/s12889-024-19475-w.

## Background

Non-communicable diseases (NCDs), namely cardiovascular disease (CVD) and diabetes, continue to be a major public health concern worldwide. CVDs constitute the leading cause of global mortality, accounting for 17.9 million deaths or nearly one third of all deaths in the world [1]. Of these deaths, approximately 85% are due to ischaemic heart disease (IHD) and stroke [1].

The global burden of CVD-related death and disability have risen over the past two decades, largely due to the combined effects of population growth, ageing, and the rising epidemic of CVD risk factors. Prevalent cases of total CVDs have increased by 93% from 271 million in 1999 to 523 million in 2019. Trends for disability-adjusted life years (DALYs) due to CVDs have also risen, with years lived with disability doubling from 17.7 million to 34.4 million over the same duration [2]. This phenomenon represents a significant challenge that must be urgently addressed as it places immense strain on healthcare systems.

Diabetes represents yet another significant driver behind the escalating burden of NCDs. An estimated 537 million adults aged 20–79 have diabetes, which translates to a global prevalence of 10.5% in this age group [3]. Diabetes has long been known as an independent risk factor for CVD and is a common precursor to a cardiovascular event. Up to one third (32.2%) of all patients with diabetes have CVD, and one in ten (9.9%) individuals with diabetes meet their demise due to CVD complications [4]. The most prevalent form of diabetes is type 2 diabetes mellitus (T2DM), which accounts for 96.0% of diabetes cases and a staggering 95.4% diabetes DALYs worldwide [5]. According to the World Health Organization, DALYs from diabetes have surged by more than 80% between 2000 and 2019 [6]. By 2050, the disease could affect more than 1.31 billion individuals and prevalence rates are predicted to surpass 20% in many parts of the world by the end of the period [5].

The burden of NCDs is especially pronounced in low- and middle-income countries (LMICs). Over three quarters of CVD deaths and more than 80% of diabetes cases occur in LMICs [1, 7]. Malaysia, a developing nation of upper-middle-income status, has the highest prevalence for diabetes in Southeast Asia [3]. Up to 3.9 million (18.3%) Malaysians aged 18–79 are affected by diabetes and more than half are unaware that they have diabetes [8]. According to the Malaysia Burden of Disease report, approximately 75% of DALYs are attributable to NCDs, with IHD, diabetes and stroke being the top three burden contributors [9, 10]. In 2017, the total direct healthcare costs for CVD and diabetes were RM 3.9 billion and RM 4.4 billion, respectively ‒ at least triple the cost for cancer (RM 1.3 billion). These included costs for hospitalisation, outpatient visits, medications, laboratory tests, allied health, and medical consumables [11].

Malaysia has a complex multiracial population represented by three predominant ethnic groups: Malay or Bumiputera (69.8%), Chinese (22.4%), and Indian (6.8%) [12]. In the national census and other forms of official administrative documentations, persons who do not fit into the three main groups are classified under a catch-all race category called “Others” (1%) [13]. This may include the Orang Asli or indigenous peoples (sometimes classified under Other Bumiputera), as well as a small number of individuals of mixed parentage, such as Eurasians and Chindians [14, 15]. Research indicates significant differences in diabetes susceptibility among different ethnic groups, with the highest prevalence seen in Indians (31.4%), followed by Malays (21.6%) and Chinese (8.5%) [8]. This variation in disease risk may be the result of genetic and lifestyle factors [16].

The health and economic burden associated with CVD in people with T2DM not only impacts affected individuals and their families, but also imposes substantial costs on healthcare providers at the societal level. To date, limited work has been done to appraise the direct treatment costs of both NCD categories combined in LMICs [17–20]. Current available data comparing financial health expenditures for CVDs in patients with and without T2DM are mainly derived from Western populations and conducted in high-income countries [21–27].

Understanding the impact of CVD on hospitalisation costs for patients with and without T2DM is crucial to inform resource allocation for disease surveillance, prevention and treatment, particularly in LMIC settings where access to healthcare services is often limited and the epidemiological burden of these conditions is substantial. To this end, we conducted a retrospective administrative database analysis to determine the hospitalisation costs incurred due to cardiovascular disease events (CVDEs) among diabetic versus non-diabetic patients in Malaysia. In addition, we sought to describe the type and incidence of CVDEs, length of stay (LoS), and CVD risk factors influencing the incremental cost of acute CVDE care in the local public health setting.

## Methods

This was a retrospective cross-sectional study using administrative data from three public tertiary hospitals (Hospital Sultan Idris Shah Serdang, Hospital Putrajaya, and Hospital Tuanku Jaafar Negeri Sembilan) in Malaysia. These hospitals were selected based on their strategic location in the central region of Peninsular Malaysia and for their large catchment areas, where a high influx of admissions related to CVDEs can be anticipated.

The primary objective of this study was to determine the hospital treatment cost for CVDEs in patients with and without T2DM. The secondary objective was to identify factors influencing treatment cost in these patients.

The primary data source was clinical and costing data extracted from the Malaysian Diagnosis Related Group (Malaysian DRG) Casemix System. A casemix system is a structured framework designed to classify patients with similar clinical characteristics and resource utilisation patterns into relatively homogeneous costing groups [28]. The most widely known example of a casemix system is the Diagnosis Related Group (DRG) classification system, where each DRG describes a cluster of patients with related diagnoses incurring similar treatment costs for an episode of care [29].

The Malaysian DRG Casemix System serves as a useful health management tool for budgeting and quality assurance monitoring [30]. To date, it has been implemented in 148 public hospitals for tracking inpatient expenditure. This system routinely collects patient variables such as patient age and sex, primary and secondary diagnoses, LoS, procedures performed, discharge status, and cost of services. Outputs generated include treatment cost per disease according to the DRG, estimated treatment cost for inpatient service care, workload metrices, and health facility efficiency index [31].

**Fig. 1:**
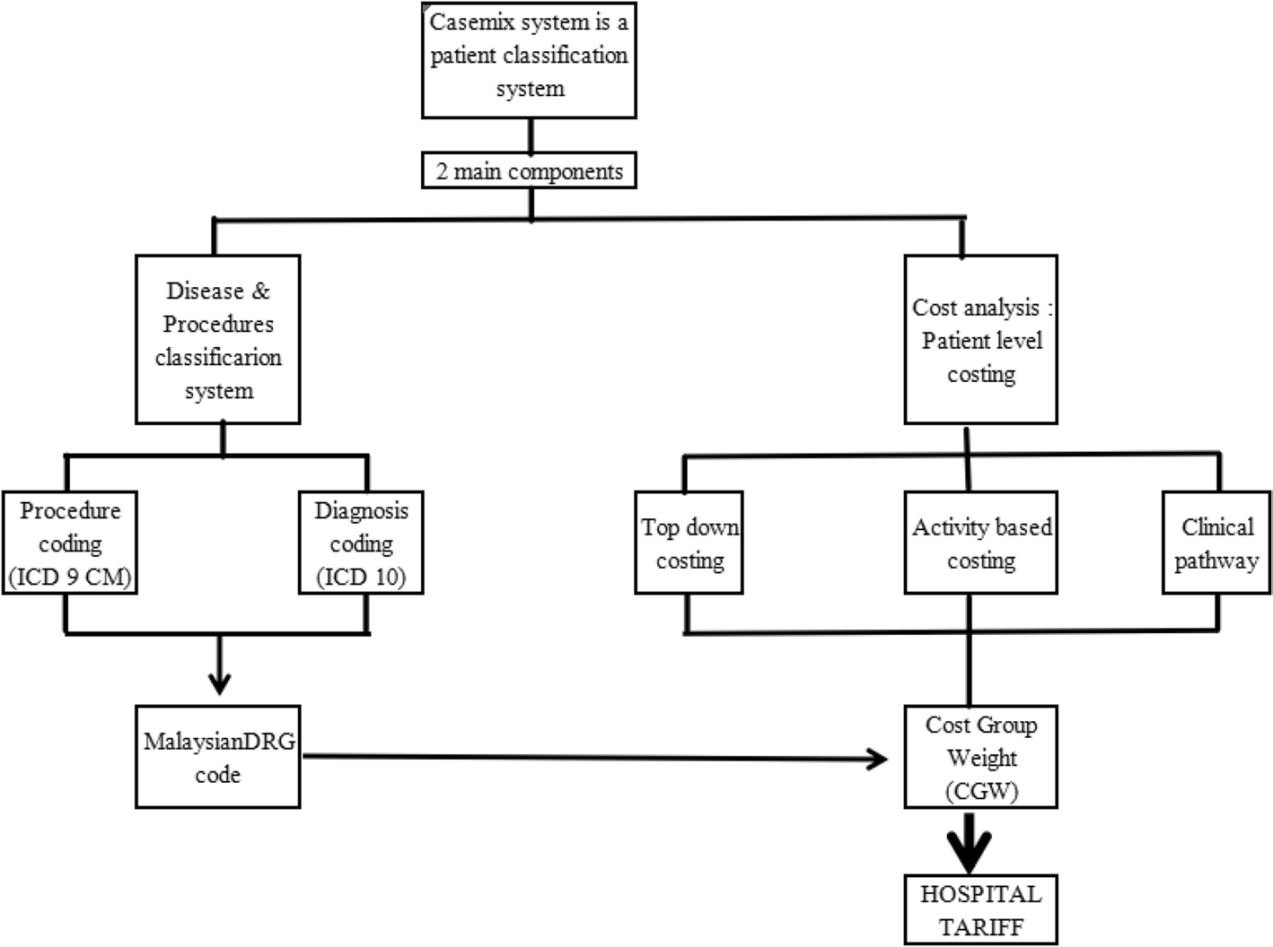
Design components of the Malaysian DRG Casemix System. ICD-10 = International Classification of Diseases 10^th^ . Revision; MY-DRG = Malaysian Diagnosis Related Group. Reproduced with permission from Zafirah et al*.* 2018 [32]

**Fig. 2:**
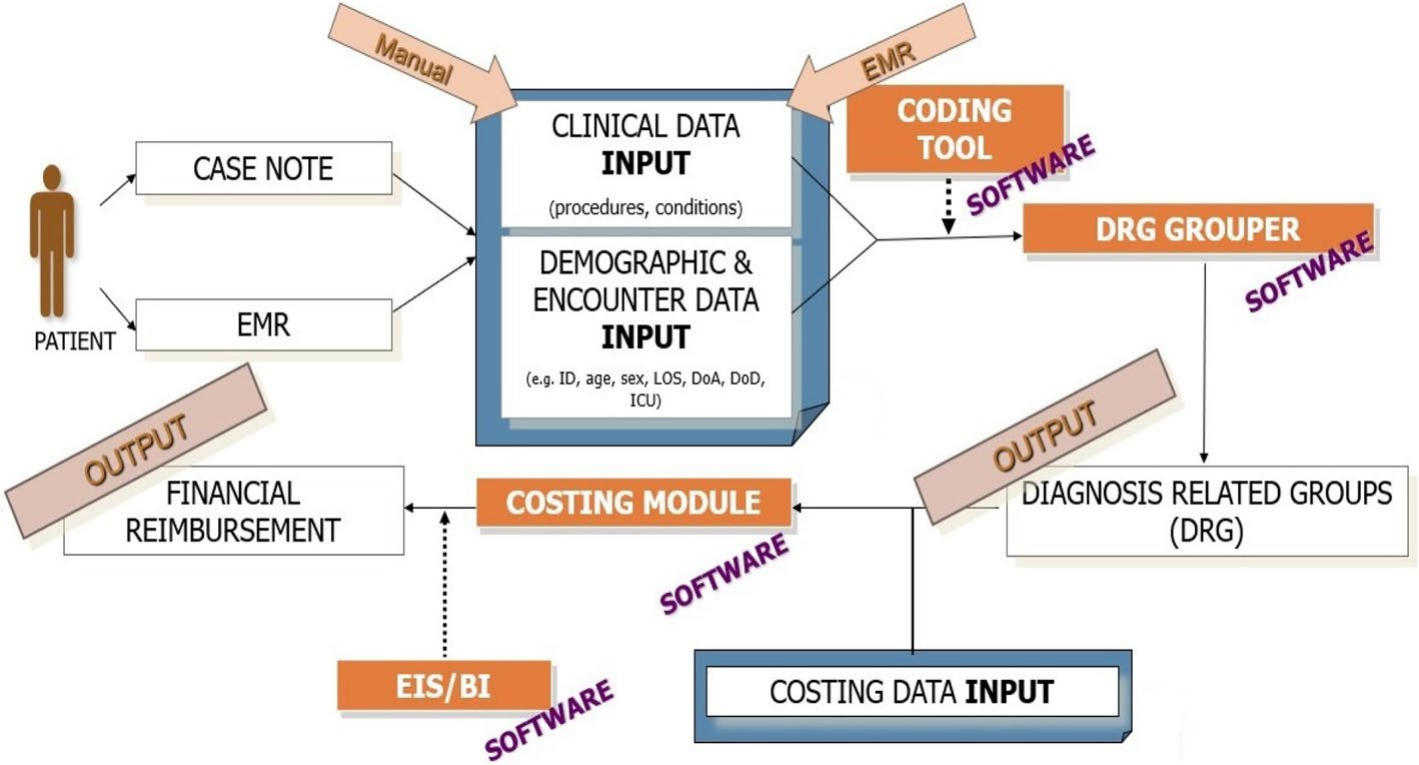
The Malaysian DRG Casemix System workflow**.** EMR = electronic medical record; EIS = Executive information system; BI = business intelligence; ID = identification; LOS = length of stay; DoA = date of admission; DoD = date of discharge; ICU = intensive care unit; DRG = diagnosis related group. Reproduced with permission from Ministry of Health Malaysia MyHEALTH Portal [33]

Figures 1 and 2 illustrate the Malaysian DRG design components and system workflow to calculate treatment costs [32, 33]. The system requires input of two important sets of information: (i) the patient’s demographic and encounter information, and (ii) clinical data [33]. When patients are discharged from the hospital, relevant information obtained from case notes generated during the episode of care are manually keyed into the system. Each patient care episode is then assigned to a DRG code. In the Malaysian DRG system, DRG codes are made up from a combination of diagnosis and procedure codes defined by the International Classification of Diseases 10th Revision (ICD-10) and International Classification 9th Revision Clinical Modification (ICD-9-CM) codes, respectively. Each CVDE is rated using a three-tiered Severity of Illness (SoI) Index (increasing in severity from Level I to III) derived based on an aggregation of health dimensions to reflect the total burden of illness and intensity of resource consumption for a patient [30]. This is determined using discharge records and scored based on the presence of complications and comorbidities, number of procedures, dependency on life support procedures, and other prognostic indicators (for example, age). A DRG code is then generated and assigned to a hospital tariff according to the cost group weight [32].

**Fig. 3:**
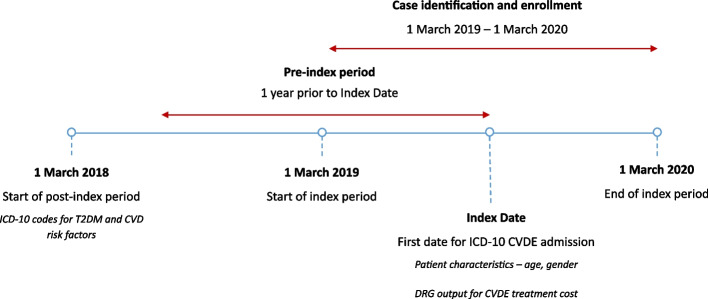
Study design and schema. ICD-10 = International Classification of Diseases 10th Revision; T2DM = type 2 diabetes mellitus; CVD = cardiovascular disease; CVDE = cardiovascular disease event; DRG = Diagnosis Related Group

Data pertaining to hospital admissions between 1 March 2019 and 1 March 2020 were retrieved from the Malaysian DRG Casemix System. The index date for each patient was defined as the date on which an ICD-10 code for a principal diagnosis of CVDE was identified. The pre-index period was defined as 12 months before the index hospital admission date. Figure 3 illustrates the study design and schema. All Malaysian patients aged ≥ 18 who were hospitalised with a principal diagnosis defined by ICD-10 codes for acute myocardial infarction (MI), IHD, hypertensive heart disease, stroke, heart failure, cardiomyopathy, and peripheral vascular disease (PVD) were included in the study (see Appendix A for ICD-10 codes). Patients were excluded if they had a history of cancer, coronavirus disease 2019 (COVID-19), hepatitis B or C, human immunodeficiency virus or major psychiatric illness. Eligible patients were assessed for T2DM status (Appendix B) and CVD risk factors (Appendix C) by extracting the relevant ICD-10 codes in the one-year pre-index period. Additional clinical information not captured by the Malaysian DRG Casemix System, such as T2DM duration and glycated haemoglobin measurement (HbA1c), were retrieved from the patient’s medical records and case notes. The DRG codes for CVDEs of interest were then extracted according to the ICD-10 codes of interest and grouped to determine the cost of treatment (Appendix D).

We used absolute numbers and percentages for categorical variables and mean ± standard deviation (SD) for continuous variables. Non-normally distributed data were presented as median and data range (minimum and maximum range). Cost analyses included cost per CVDE and total cost per year for CVDEs. All costs are expressed in Malaysian Ringgit (RM) for the financial year FY2020 from 1 March 2019 to 1 March 2020 (average exchange rate is 1 USD = RM 4.20 for 2020). Patients were stratified and analysed according to T2DM status. As treatment costs did not conform to normal distribution, we used the Mann Whitney test for inter-group comparison between T2DM and non-T2DM patients. A Chi-square test of independence was conducted to determine the relationship between independent variables and cost. A median cost were calculated for CVDE among T2DM and non-T2DM patients. Multivariate analysis using binary logic regression was used to determine predictors of high CVDE cost among T2DM and non-T2DM patients. A median cost for CVDE among T2DM were used for this analysis. Data analyses were conducted using Microsoft Excel and SPSS Software version 26.0. A *P* value of < 0.05 was considered statistically significant.

## Results

A total of 4,643 admission records between 1 March 2019 and 1 March 2020 with CVDE as the principal diagnosis were identified from the Malaysian DRG Casemix System from these three hospitals. Using random sampling technique, we selected and screened 1192 patients for eligibility. Of these, a final sample size of 1,183 patients were included for analyses. Table 1 provides an overview of the patients’ demographic and clinical characteristics. Overall, the mean age of patients admitted for CVDE was 58.6 years. The youngest patient was 18 years old while the oldest patient was 91 years old. The most common CVDE diagnosis was acute MI (25.6%), followed by IHD (25.3%), hypertensive heart disease (18.9%), stroke (12.9%) and heart failure (9.4%). CVDE admissions were less common for cardiomyopathy (5.7%) and PVD (2.1%). The mean (average) LoS was 4.8 days. A longer average LoS was observed in patients with more complex illness (5.6 days for Severity III versus 4.4 days for Severity I). Nearly two-thirds (62.4%) of all patients who were admitted for a CVDE had at least one CVD risk factor. The most frequent risk factor was hypertension (60.4%), followed by PVD (17.8%), dyslipidaemia (10.1%) and previous stroke (6.9%).

**Table 1:** Patient demographic and clinical characteristics

		**CVDE patients**	**CVDE patients with T2DM**	**CVDE patients without T2DM**
Total, N (%)		1183 (100)	715 (60.4)	468 (39.6)
Age, mean (years ± SD)		58.6 ± 13.2	59.1 ± 12.8	58.0 ± 13.7
Gender				
Male		778 (65.8)	425 (59.4)	353 (75.4)
Female		405 (32.2)	290 (40.6)	115 (24.6)
Ethnicity				
Malay		763 (64.5)	487 (68.1)	276 (59.0)
Chinese		185 (15.6)	80 (11.2)	105 (22.3)
Indian		235 (19.9)	148 (20.7)	87 (18.6)
Age category (years)				
18‒29		18 (1.5)	14 (2.0)	4 (0.9)
30‒39		83 (7.0)	50 (7.3)	31 (6.6)
40‒49		184 (15.6)	113 (15.8)	71 (15.2)
50‒59		312 (26.4)	176(24.6)	136 (29.1)
60‒69		336 (28.4)	207 (29.0)	129 (27.6)
≥ 70		250 (21.1)	153 (21.4)	97 (20.7)
Type of CVDE				
Acute MI		303 (25.6)	151 (21.1)	152 (32.5)
IHD		300 (25.3)	129 (18.0)	171 (36.5)
Hypertensive HD		224 (18.9)	186 (26.0)	3.8 (8.1)
Stroke		153 (12.9)	91 (12.7)	62 (13.2)
Heart failure		111 (9.4)	91 (12.7)	20 (4.3)
Cardiomyopathy		67 (5.7)	47 (6.6)	20 (4.3)
PVD		25 (2.1)	20 (2.8)	5 (1.1)
Outcome of admission				
Discharged well		1113 (94.1)	662 (92.6)	451 (96.4)
Death		70 (5.9)	53 (7.4)	17 (3.6)
Severity level				
Severity I		306 (26.0)	174 (24.3)	134 (28.6)
Severity II		530 (44.8)	354 (49.5)	176 (37.6)
Severity III		345 (29.2)	187 (26.2)	158 (33.8)
Average LoS (days ± SD)				
Severity I		4.71 ± 3.51	4.87 ± 3.72	3.85 ± 1.93
Severity II		5.06 ± 3.85	5.22 ± 3.98	3.7 ± 2.00
Severity III		6.98 ± 7.38	6.68 ± 6.9	8.07 ± 8.99
Presence of CVD risk				
Yes		738 (62.4)	453 (63.4)	285 (60.9)
No		445 (37.6)	262 (36.6)	183 (39.1)
Number of CVD risks				
1		644 (54.4)	406 (56.9)	238 (50.7)
2		91 (7.7)	46 (6.4)	45 (9.6)
≥ 3		12 (1)	7 (1.0)	5 (1.1)
Type of CVD risk				
Hypertension		714 (60.4)	482 (67.5)	232 (49.5)
Dyslipidaemia		119 (10.1)	62 (8.6)	57 (12.2)
PVD		211 (17.8)	210 (29.4)	1 (0.3)
Stroke		82 (6.9)	60 (8.4)	22 (5.9)

All variables are reported as frequency and percentage unless otherwise specified.

*CVDE *cardiovascular disease event, *IHD *ischaemic heart disease, *MI *myocardial infarction, *HD *heart disease, *PVD *peripheral vascular disease, *LoS *length of stay, *T2DM *type 2 diabetes mellitus, *SD *standard deviation

### Incidence of CVDE in T2DM and non-T2DM patients

Approximately 60.4% of patients with CVDE had underlying T2DM. Compared with non-T2DM patients, the cohort with T2DM were slightly older, were comprised of more women and had a higher prevalence of CVD risk factors (Table 1). Nearly half (48.7%, n = 348/714) of patients with T2DM had a duration of diabetes of ≤ 5 years. Data for HbA1c was available for 351 patients. Three quarters of these patients (75.2%) had a HbA1c greater than 7.0%. The proportion of patients with T2DM who did not survive their admission was twice as high compared with those without T2DM (7.4% versus 3.2%, respectively).

We found differing CVDE frequencies between T2DM and non-T2DM patients. Within the T2DM group, the most common type of CVDE was hypertensive heart disease (26.0%), followed by acute MI (21.1%) and IHD (18.0%). In the non-T2DM group, the predominant type of CVDE was IHD (36.5%), followed by acute MI (32.5%) and stroke (13.2%). Over 60% of patients had at least one CVD risk factor, with hypertension being the most prevalent in both the T2DM and non-T2DM groups. Patients with T2DM were more frequently affected by PVD (29.4%) as opposed to those without T2DM (0.3%). In contrast, patients without T2DM (12.2%) were more likely to have dyslipidaemia compared with their T2DM counterparts (8.4%).

### Cost of CVDE in T2DM and non-T2DM patients

Table 2 shows the total and individual costs for CVDE treatment in T2DM and non-T2DM patients. The overall expenditure (total cost for all cases) for inpatient CVDE treatment was approximately RM 8.4 million. Patients with T2DM incurred a higher cost in excess of RM 1.1 million, about 30% higher than the amount incurred by patients without T2DM. Despite the higher overall cost incurred by T2DM patients, the median cost per case (or cost per episode of CVDE care) was slightly lower (RM 5,452.63) compared to non-T2DM patients (RM 6,941.30). IHD incurred the highest cost: up to RM 1.4 million in the T2DM group and RM 1.8 million in the non-T2DM group. This constituted 30.0% and 50.0% of the total CVDE treatment cost for patients with and without T2DM, respectively. The median cost per case for IHD was RM 8,364.65, more than double the cost for acute MI or stroke. The higher cost for IHD could be attributed to resource-intensive procedures, such as percutaneous coronary intervention and coronary bypass grafting, resulting in increased average LoS and cost per case for the provider (Table 3).

**Table 2:** Cost of CVDE treatment in T2DM and non-T2DM patients for FY2020

	**CVDE with T2DM**					**CVDE without T2DM**				
**Diagnosis**	**N**	**Median cost per case (RM)**	**Minimum cost (RM)**	**Maximum cost (RM)**	**Total cost for all cases (RM)**	**N**	**Median cost per case (RM)**	**Minimum cost (RM)**	**Maximum cost (RM)**	**Total cost for all cases (RM)**
Acute MI	151	4,608.38	2,989.98	16,373.72	874,388.08	152	4,649.53	2,989.98	14,197.31	941,588.78
IHD	129	8,364.65	3,235.20	106,357.88	1,415,411.24	171	8,364.65	3,545.80	106,357.88	1,837,803.52
Hypertensive HD	186	5,440.91	2,573.20	9,946.92	1,021,690.43	38	4,740.02	2,789.78	9,477.31	206,964.00
Stroke	91	4,521.59	2,989.98	100,790.23	582,047.77	62	4,521.59	2,989.98	9,860.14	314,731.73
Heart failure	91	5,440.91	2,573.20	17,462.31	495,503.89	20	5,756.94	3,954.56	100,790.23	206,582.14
Cardiomyopathy	47	5,452.63	3,827.03	14,197.31	276,592.20	20	6,371.82	3,147.64	10,847.26	127,034.15
PVD	20	5,090.46	3,472.18	14,943.43	119,962.18	6	5,149.68	3,235.20	8,364.65	30,898.08
Total	715	5,452.63	2,573.20	106,357.88	4,785,595.79	468	6,941.30	2,789.78	106,357.88	3,658,555.47

*CVDE *cardiovascular disease event, *T2DM *type 2 diabetes, *FY *financial year, *MI *myocardial infarction, *IHD *ischaemic heart disease, *HD *heart disease, *PVD *peripheral vascular disease

**Table 3:** Cost per case (CPC) and average length of stay (LoS) per DRG code

DRG code	DRG details	Cases, n (%)	CPC (RM)	Average LoS (days)
**Cardiovascular Disease Events (n = 614)**				
	**Coronary artery disease**	228 (19.3)		
5531	Without complication	69 (30.3)	3,954.56	3.80
5532	With complication	128 (56.2)	4,521.59	4.00
5533	With major complication	31 (13.5)	7,121.32	5.20
	**Vascular disorder and injuries**	105 (8.9)		
5611	Without complication	34 (32.3)	5,440.91	5.50
5612	With complication	63 (60.0)	5,708.21	5.80
5613	With major complication	8 (7.7)	8,672.86	8.70
	**Heart failure and shock**	94 (7.9)		
5571	Without complication	30 (31.9)	4,740.02	4.50
5572	With complication	3 (3.2)	4,690.69	4.60
5573	With major complication	61 (64.9)	5,805.67	5.60
	**Nonspecific cerebrovascular disorder**	63 (5.3)		
1651	Without complication	15 (23.8)	4,608.38	4.70
1652	With complication	35 (55.5)	5,452.63	5.70
1653	With major complication	13 (20.6)	8,153.81	8.50
	**Ischaemic cerebrovascular disease**	59 (4.9)		
1551	Without complication	7 (11.8)	2,989.98	3.60
1552	With complication	39 (66.1)	3,235.20	3.90
1553	With major complication	13 (22.0)	6,278.19	7.80
	**Atherosclerosis**	21 (1.8)		
5521	Without complication	13 (61.9)	6,898.62	3.50
5522	With complication	5 (23.8)	6,928.42	3.80
5523	With major complication	3 (14.2)	11,008.35	5.30
	Myocardial disease	19 (1.6)		
5541	Without complication	4 (21.0)	6,889.68	4.70
5542	With complication	10 (52.6)	6,941.30	4.60
5543	With major complication	5 (26.4)	9,477.31	6.80
	**Cardiac arrythmia and conduction disorder**	11 (0.9)		
5561	Without complication	3 (27.3)	3,722.52	3.10
5562	With complication	6 (54.5)	4,598.89	3.80
5563	With major complication	2 (18.2)	5,960.91	4.80
	**Cardiac arrest, unexplained**	9 (0.8)		
5593	With major complication	9 (100)	9,389.25	8.30
	**Hypertension**	5 (0.4)		
5511	Without complication	1 (20.0)	2,849.30	3.30
5512	With complication	1 (20.0)	3,168.96	3.60
5513	With major complication	3 (60.0)	4,342.06	4.80
**Procedures related to CVDE (n = 341)**				
	**Percutaneous coronary intervention**	315 (26.6)		
5041	Without complication	57 (18.0)	7,753.10	3.20
5042	With complication	103 (32.7)	8,364.65	3.50
5043	With major complication	155 (49.3)	9,860.14	3.60
	**Coronary bypass procedure**	10 (0.8)		
5001	Without complication	2 (20.0)	106,357.88	16.80
5002	With complication	1 (10.0)	102,400.48	18.30
5003	With major complication	7 (70.0)	100,790.23	22.70
	**Electrophysiology study or RFA and pacemaker insertion**	5 (0.3)		
5062	With complication	2 (40.0)	10,847.26	4.20
5063	With major complication	3 (60.0)	14,197.31	5.20
	**Other circulatory system operating room procedure**	3 (0.2)		
5101	Without complication	1 (33.3)	3,391.62	3.50
5102	With complication	2 (66.7)	4,510.08	4.30
	**Other vascular procedure**	4 (0.2)		
5091	Without complication	1 (25.0)	3,582.61	3.80
5092	With complication	2 (50.0)	4,749.74	4.00
5093	With major complication	1 (25.0)	11,275.83	9.30
	**Spinal cord and spinal canal procedure**	1 (0.1)		
1051	Without complication	1 (100.0)	7,740.05	8.60
	Vein ligation and stripping	1 (0.1)		
5081	Without complication	1 (100.0)	4,203.07	2.90
	**Other respiratory system operating room procedure**	2 (0.1)		
4011	Without complication	1 (50.0)	6,853.90	6.70
4013	With major complication	1 (50.0)	21,649.81	18.40

*DRG *Disease Related Group, *CVDE*  cardiovascular disease event, *RFA *radiofrequency ablation

For the T2DM group, median per case showed an incremental increase with each additional CVD risk factor (Table 4). On the other hand, cost per case was highest in non-T2DM patients with two CVD risk factors and lowest for those with three or more risk factors. The cost per case for each CVD risk factor of interest can be found in Table 5. The median cost per case when hypertension and hyperlipidaemia were present were similar in both groups. However, a higher median cost per case was incurred by patients with T2DM if they also had a history of prior IHD or concomitant PVD.

**Table 4:** Cost of CVDE management by number of CVD risk factors

Number of CVD risk factors	CVDE with T2DM				CVDE without T2DM			
	**N**	**Median cost (RM)**	**Minimum cost (RM)**	**Maximum cost (RM)**	**N**	**Median cost (RM)**	**Minimum cost (RM)**	**Maximum cost (RM)**
1	429	4,376.78	2,341.75	66,413.89	183	4,376.78	2,531.11	53,757.11
2	45	7,118.00	2,683.25	9,970.46	45	7,118.00	2,683.25	860,411.00
≥ 3	7	8,604.11	4,232.30	53,757.11	5	2,683.25	2,508.80	7,118.00

*CVDE *cardiovascular disease event, *CVD *cardiovascular disease, *T2DM*  type 2 diabetes

**Table 5:** Cost of CVDE treatment in T2DM and non-T2DM patients with cardiovascular risks of interest

	**CVDE with T2DM**				**CVDE without T2DM**			
**Cardiovascular risk**	**N**	**Median cost (RM)**	**Minimum cost (RM)**	**Maximum cost (RM)**	**N**	**Median cost (RM)**	**Minimum cost (RM)**	**Maximum cost (RM)**
Hypertension	482	4,377.00	2,342.00	66,414.00	233	4,743.44	2,509.00	53,757.00
Hyperlipidaemia	151	7,118.00	2,683.00	53,757.00	57	7,118.00	2,509.00	8,604.00
IHD	180	9,173.98	2,738.71	68,685.24	137	8,528.57	3,119.45	53,757.11
Stroke	60	4,363.76	2,509.00	53,757.00	NA	NA	NA	NA
PVD	210	4,405.31	2,342.00	8,604.00	917	2,907.44	2,907.00	2,907.00

*CVDE *cardiovascular disease event, *T2DM *type 2 diabetes, *IHD *ischaemic heart disease, *PVD *peripheral vascular disease, *NA *not available

### Factors influencing cost of CVDE treatment

The median CVDE cost calculated for patients with T2DM (RM 5,452.63) was used to as the threshold for categorising treatment cost level (low versus high). As shown in Table 6, the cost of CVDE treatment was significantly associated with gender, outcome of admission, type of CVDE, SoI Index level, T2DM status, and the presence of CVD risk factors. Age, ethnicity, duration of diabetes, and HbA1c level did not significantly influence treatment costs. Certain factors were significantly correlated with CVDE treatment cost in patients with T2DM patients (Table 7). These factors included male gender, age, admission for IHD, outcome of admission, and the presence of CVD risk. For patients without T2DM, only two factors significantly correlated with treatment costs: the type of CVDE and the SoI Index level.

**Table 6:** Association between patient characteristics and total cost (*N* = 1183)

Variable		Cost		X^2^	df	*p*-value
		**Low, n (%)**	**High, n (%)**			
Gender				7.863	1	0.005*
Male		316 (40.6)	462 (59.4)			
Female		199 (49.1)	206 (50.9)			
Ethnicity				5.64	2	0.06
Malay		350 (45.9)	413 (54.1)			
Chinese		68 (36.8)	117 (63.2)			
Indian		97 (41.3)	138 (58.7)			
Age category (years)				7.09	5	0.214
18‒29		10 (55.6)	8 (44.4)			
30‒39		41 (49.4)	42 (50.6)			
40‒49		70 (38.0)	114 (62.0)			
50‒59		126 (135.8)	186 (59.6)			
60‒69		151 (44.9)	185 (55.1)			
≥ 70		117 (46.8)	133 (53.2)			
Type of CVDE				186.593	6	< 0.001*
Acute MI		162 (53.5)	141 (46.5)			
IHD		31 (10.3)	269 (89.7)			
Hypertensive heart disease		118 (52.7)	106 (47.3)			
Stroke		95 (66.6)	58 (37.9)			
Heart failure		63 (56.8)	48 (43.2)			
Cardiomyopathy		31 (46.3)	36 (53.7)			
Peripheral vascular disease		15 (60)	10 (40.0)			
Outcome of admission				11.213	1	0.001*
Discharged well		498 (44.7)	615 (55.3)			
Death		17 (24.3)	53 (75.7)			
Severity level				358.01	2	< 0.001*
Severity I		215 (69.8)	93 (30.2)			
Severity II		293 (55.3)	237 (44.7)			
Severity III		7 (2.0)	338 (98.0)			
Diabetes status				11.064	1	0.001*
Yes		339 (65.8)	376 (56.3)			
No		176 (34.2)	292 (43.7)			
Diabetes duration (years)				8.199	4	
0‒5		166 (47.7)	182 (52.3)			
6‒10		55 (42.0)	76 (58.0)			
11‒15		59 (48.4)	63 (51.6)			
16‒20		42 (54.5)	35 (45.5)			
≥ 20		24 (66.7)	12 (33.3)			
HbA1c level (%)				4.554	3	0.206
0.0‒6.5		28 (48.3)	30 (51.7)			
6.6‒7.0		15 (11.6)	14 (17.4)			
7.1‒8.0		19 (21.5)	35 (64.8)			
≥ 8.0		78(37.1)	132 (62.9)			
Presence of CV risk factor				21.469	1	< 0.001*
Yes		283 (55.0)	455 (68.1)			
No		232 (45.0)	213 (31.9)			

*CVDE *cardiovascular disease event, *MI *myocardial infarction, *IHD *ischaemic heart disease, *HbA1c *glycated haemoglobin, *CV *cardiovascular

**Table 7:** Association between patient characteristics and cost among T2DM and non-T2DM patients (*N* = 1183)

Variables		CVDE with T2DM (n = 715)					CVDE without T2DM (n = 496)				
		**Cost, n (%)**		**X**^**2**^	**df**	***P* value**	**Cost**		**X**^**2**^	**df**	***P* value**
		**Low**	**High**				**Low**	**High**			
Gender				2.101	1	0.147			3.764	1	0.052
Male		192 (56.6)	233 (62.0)				124 (70.5)	229 (78.4)			
Female		147 (43.4)	143 (38.0)				52 (29.5)	63 (21.6)			
Ethnicity				6.385	3	0.094			1.739	3	0.649
Malay		235 (69.3)	245 (65.2)				108 (61.4)	166 (56.8)			
Chinese		34 (10.0)	46 (12.2)				34 (19.3)	71 (24.3)			
Indian		64 (18.9)	84 (22.3)				33 (18.8)	54 (18.5)			
Others		6 (1.8)	1 (0.3)				1 (0.6)	1 (0.3)			
Age category (years)				18.477	5	0.002*			5.747	5	0.332
18‒29		16 (4.7)	5 (1.3)				5 (2.8)	2 (0.7)			
30‒39		33 (9.7)	15 (4.0)				22 (12.5)	28 (9.6)			
40‒49		41 (12.1)	61 (16.2)				35 (19.9)	58 (19.9)			
50‒59		94 (27.7)	111 (29.5)				39 (22.2)	81 (27.7)			
60‒69		100 (29.5)	120 (31.9)				42 (23.9)	72 (24.7)			
≥ 70		55 (16.2)	64 (17.0)				33 (18.8)	51 (17.5)			
Type of CVDE				83.717	6	< 0.001			98.463	6	< 0.001
Acute MI		84 (24.8)	67 (17.8)				78 (44.3)	74 (25.3)			
IHD		15(4.4)	114 (30.3)				16 (9.1)	155 (53.1)			
Hypertensive HD		97(28.6)	89 (23.7)				21 (11.9)	17 (5.8)			
Stroke		54 (15.9)	37 (9.8)				41 (23.3)	21 (7.2)			
Heart failure		54 (15.9)	37 (9.8)				9 (5.1)	11 (3.8)			
Cardiomyopathy		23 (6.8)	47 (6.6)				8 (4.5)	12 (4.1)			
PVD		12 (3.5)	20 (2.9)				3 (1.7)	2 (0.7)			
Outcome of admission				12.023	1	0.001*			1.490	1	0.222
Discharged well		326 (96.2)	336 (89.4)				172 (97.7)	279 (95.5)			
Death		13 (3.8)	40 (10.6)				4 (2.3)	13 (4.5)			
Severity level				201.77	2	< 0.001*			136.836	2	< 0.001*
Severity I		133 (39.2)	41 (10.9)				82 (46.6)	52 (17.8)			
Severity II		201 (59.3)	153 (40.7)				92 (52.3)	84 (28.8)			
Severity III		5 (1.5)	182 (48.4)				2 (1.1)	156 (53.4)			
Presence of CVD risk factor				29.226	1	< 0.001*					
Yes		180 (53.1)	273 (72.6)								
No		159 (46.9)	103 (27.4)								
Duration of diabetes (years)				6.869	4	0.231					
0‒5		161 (47.5)	192 (51.1)								
6‒10		61 (18.0)	81 (21.5)								
11‒15		60 (17.7)	58 (15.4)								
16‒20		28 (8.3)	27 (28.9)								
≥ 20		28 (8.3)	18 (4.8)								
HbA1c level (%)				4.502	3	0.217					
0.0‒6.5		15 (17.4)	21 (12.8)								
6.6‒7.0		10 (11.6)	9 (5.5)								
7.1‒8.0		11 (12.8)	23 (14.0)								
≥ 8.0		50 (58.1)	111 (67.7)								

*CVDE *cardiovascular disease event, *T2DM *type 2 diabetes, *MI *myocardial infarction, *IHD *ischaemic heart disease, *HD *heard disease, *PVD *peripheral vascular disease, *CVD *cardiovascular disease, *HbA1c *glycated haemoglobin

Using the median cost of CVDE among T2DM (RM 5,452.63) as a threshold for high versus low cost, multivariate logistic regression analysis was performed. Initially, nine variables including age, sex, ethnicity, type of CVDE, SoI Index level, and CVD risk were included in the model. Among these, gender, ethnicity, type of CVDE, and SoI Index level were found to be significant predictors of high treatment costs for CVDE (Table 8).

**Table 8:** Multivariate logistic regression analysis

		**β**	**SE**	**Wald**	**Adjusted odds ratio**		**95% CI**		***P* value**
							**Lower**	**Upper**	
**Gender**									
Male					1				
Female		-0.416	0.174	5.686	0.660		0.469	0.929	0.017
**Ethnicity**									
Malay				9.315	1				0.025
Chinese		0.333	0.245	1.848	1.395		0.863	2.256	0.174
Indian		0.036	0.216	0.028	1.037		0.679	1.585	0.867
Others		-3.461	1.275	7.363	0.031		0.003	0.382	0.007
**Severity level**									
Level I				128.509	1				< 0.01
Level II		1.006	0.197	26.09	2.734		1.859	4.022	< 0.01
Level III		5.57	0.497	125.842	262.405		99.16	694.397	< 0.01
**Type of CVDE**									
Acute MI				125.293	1				< 0.01
IHD		2.49	0.258	93.174	12.056		7.272	19.987	< 0.01
Hypertensive HD		-0.035	0.248	0.019	0.966		0.594	1.570	0.889
Stroke		-0.461	0.264	3.051	0.631		0.376	1.058	0.081
Heart failure		-0.047	0.314	0.022	0.954		0.515	1.767	0.881
Cardiomyopathy		0.438	0.345	1.614	1.550		0.788	3.048	0.204
PVD		0.005	0.54	0	1.005		0.349	2.899	0.992

*SE*  standard error, *CI *confidence interval, *CVDE *cardiovascular disease event, *MI *myocardial infarction, *IHD *ischaemic heart disease, *HD *heart disease, *PVD *peripheral vascular disease

The analysis revealed that females were less likely to incur high CVDE costs compared to males (odds ratio [OR] = 0.66; 95% confidence interval [CI] 0.47‒0.93, P = 0.017), indicating a statistically significant association. Furthermore, patients of 'Others' ethnicity had significantly lower odds of incurring high treatment costs for CVDE compared to Malay ethnicity (OR = 0.03; 95% CI 0.003‒0.382, P = 0.007). The SoI Index level III (high disease burden) showed a substantially increased risk (OR = 262) of incurring high costs compared to severity level I, while severity level II (moderate disease burden) showed a more modest increase (OR = 2).

In discussing these results, the significance of the β coefficients (representing the effect size), standard errors (SE), and Wald values (indicative of the significance of each predictor) should be noted. β coefficients of gender and ethnicity that negative value provide the less likelihood direction and positive value for severity level and type of CVDE indicate likelihood of incurring high CVDE costs. Standard errors reflect the precision of these estimates, with smaller SE values indicating more reliable estimates. Wald values assess the significance of each predictor, with P-values less than 0.05 suggesting stronger evidence against the null hypothesis.

Overall, these findings underscore the importance of demographic and clinical factors in determining the economic burden of CVDE treatment among T2DM patients.

## Discussion

This retrospective, cross-sectional study conducted at three public tertiary hospitals in Malaysia provides evidence regarding the epidemiological, clinical, and economic impact of CVDEs in hospitalised patients with and without T2DM. The Malaysian healthcare system is organised as a two-tiered system, consisting of a tax-funded public sector and a fee-for-service private sector [34]. The former provides universal health coverage through a network of government health facilities that caters to the bulk (~ 65%) of the population [35]. Public healthcare is heavily subsidised by the government, with patients paying a nominal fee for inpatient and outpatient services [36]. For example, patients are only charged RM 3 (~ USD 0.70, USD 1 = RM 4.20) for a third-class ward, inclusive of inpatient treatment and ward fees. Sustainability of this healthcare system relies on proficient fiscal management to maintain affordability and quality of care.

In our study, T2DM was present in 60.4% of CVDE patients as in Table 1. This figure was higher than those reported in most studies [37–43]. Available data indicate a wide range of diabetes prevalence among patients with CVD, between 20‒30% in the Western countries [37], 20‒60% in China, India and Southeast Asia [38–43], and nearly 70% in the Middle East [44]. The reasons for the large proportion of patients with T2DM in our cohort may be due to the selection of patients from hospitals in urbanised areas, where risk factors for developing T2DM such as sedentary lifestyle, unhealthy diets, and obesity are prevalent.

The mortality rate among CVDE patients with T2DM in our cohort was twice as high as for patients without T2DM. Previous studies have consistently reported an elevated risk of incident CVDs and premature deaths in patients with T2DM, and these risks are amplified when patients have a history of both T2DM and prior CVDE, in contrast to those with T2DM or prior CVDE alone [21, 45–47]. As such, patients with T2DM who have survived a CVDE constitute a particularly vulnerable to recurrent events and increased healthcare expenditure [21]. In Tables 4 and 5, this study demonstrates that the median cost increases as the number of risk factors for CVDE among T2DM populations increases, from one risk factor to three. The risk associated with a previous episode of IHD resulted in the highest median cost compared to other risk factors such as hypertension, hyperlipidemia, stroke, and peripheral vascular disease (PVD). Implementing secondary prevention strategies that prioritise intensified cardioprotective interventions are imperative for these patients.

Studies have shown that patients with diabetes consume more healthcare resources [48–50], and incur 2.3 times more in hospitalisation cost than the general population [50]. In our T2DM cohort, total treatment cost for all CVDEs exceeded those of the non-T2DM group but the median cost per case in patients with T2DM was lower than in non-T2DM patients as shown in Tables 2 and 3. This may be explained by the high proportion of T2DM patients with recently diagnosed or early-stage diabetes, where almost half of the T2DM cohort had a disease duration of ≤ 5 years. The non-T2DM group were pre-dominantly male and had a greater proportion of patients with severity Level III illness compared with the T2DM cohort.

We identified four variables predicting high treatment cost for CVDEs as stated in Table 7 and 8. Patients with the following profile are likely to incur treatment costs in excess of the median threshold: male gender, non-minority ethnicity (Malay, Chinese and Indian), IHD diagnosis, and moderate-to-severe SoI (Levels II and III). The gender differences in CVDE treatment costs may be attributed to biological and behavioural factors affecting predisposition and disease onset [51]. Premenopausal women experience a higher degree of cardioprotection than men of similar age and have a more favourable blood pressure and lipid profile. In addition to that, women are more inclined to exhibit behaviours that lower the risk of CVDs. Studies indicate that they are more likely than men to be non-smokers [52], abstain from or drink less alcohol [53], and adopt healthy eating habits [54]. They also have higher participation in preventive health checks for CVDs and are more likely to seek care early in the disease process [55, 56].

We acknowledge several limitations inherent to the design of our study. Administrative data sources are prone to coding errors, which can lead to incorrect assignment of the DRG codes and inaccurate cost estimations. We have taken steps to address these limitations by selecting audited sites, and have used additional data sources, such as patient medical records and case notes, to ensure a sufficient level of clinical data. In the present study, only costs per episode of care were examined. Therefore, we are unable to draw conclusions regarding lifetime costs or outcomes which will be done from longitudinal data over years. Last but not least, although our dataset was drawn from a demographically diverse, multicentre cohort, our results have limited generalisability. Patients admitted to tertiary hospitals typically require specialised and complex care, so our findings may not be representative of the treatment cost across all of Malaysia.

## Conclusion

This study provides real-world cost estimates for CVDE hospitalisation and quantifies the combined burden of two major NCDs categories at the public health provider level. Results confirm that CVDs are associated with substantial health utilisation in both T2DM and non-T2DM patients. Additional allocation of resources for intensified and targeted public health interventions may be justified to reduce CVD risk factors and to contain public health expenditure. The findings from this study may be used for future health technology assessments and economic modelling.

### Supplementary Information

Supplementary Material 1.

Supplementary Material 2.

Supplementary Material 3.

Supplementary Material 4.

## Data Availability

The datasets analysed during this study are available from the corresponding author on reasonable request.

## Abbreviations

BI: Business intelligence
CI: Confidence interval
CPC: Cost per case
COVID-19: Coronavirus disease 2019
CV: Cardiovascular
CVD: Cardiovascular disease
CVDE: Cardiovascular disease event
DALY: Disability-adjusted life year
DoA: Date of admission
DoD: Date of discharge
DRG: Diagnosis Related Group
EIS: Executive information system
EMR: Electronic medical record
FY: Financial year
HbA1c: Glycated haemoglobin
HD: Heart disease
ICD-9-CM: International Classification of Diseases, 9th Revision, Clinical Modification
ICD-10: International Classification of Diseases, 10th Revision
ICU: Intensive care unit
ID: Identification
IHD: Ischaemic heart disease
LMIC: Low- and middle-income country
LoS: Length of stay
MI: Myocardial infarction
MY-DRG: Malaysia Diagnosis Related Group
NA: Not available
NCD: Non-communicable disease
OR: Odds ratio
PVD: Peripheral vascular disease
RFA: Radiofrequency ablation
RM: Malaysian Ringgit
SD: Standard deviation
SE: Standard error
SoI: Severity of illness
T2DM: Type 2 diabetes mellitus
USD: United States Dollar
