# Supplementary material for: Cost of cardiovascular disease events in patients with and without type 2 diabetes and factors influencing cost: a retrospective cohort study
Source: BMC Public Health. 2024 Jul 26;24:2003. doi: 10.1186/s12889-024-19475-w (PMC11282681; doi:10.1186/s12889-024-19475-w)
Supplement: Supplementary file 1 — Supplementary Material 1. [file 12889_2024_19475_MOESM1_ESM.docx]

**Appendix A. ICD-10 codes used to identify cases of cardiovascular events**

| **Definition** | | **ICD-10 codes** | |
| --- | --- | --- | --- |
| **Hypertensive heart disease** | |  |  |
|  | Hypertensive heart disease |  | I11 |
|  | Hypertensive heart disease with heart failure |  | I11.0 |
|  | Hypertensive heart disease without heart failure |  | I11.9 |
| **Hypertensive heart and renal disease** | |  |  |
|  | Hypertensive heart disease and hypertensive renal disease |  | I13 |
| **Ischaemic heart disease** | |  |  |
|  | Angina pectoris |  | I20 |
|  | Acute myocardial infarction |  | I21 |
|  | Subsequent ST-elevation (STEMI) and non-ST-elevation myocardial infarction (NSTEMI) |  | I22 |
|  | Certain current complications following STEMI and NSTEMI (within the 28-day period) |  | I23 |
|  | Other acute ischaemic heart diseases |  | I24 |
|  | Chronic ischaemic heart diseases |  | I25 |
| **Cardiomyopathy** | |  |  |
|  | Cardiomyopathy – diseases of cardiac muscle |  | I42.0 |
| **Cardiac arrest** | |  |  |
|  | Cardiac arrest |  | I46 |
| **Other cardiac arrhythmias** | |  |  |
|  | Ventricular fibrillation and flutter |  | I49.0 |
|  | Ventricular fibrillation |  | I49.01 |
|  | Ventricular flutter |  | I49.02 |
|  | Unspecific cardiac dysrhythmias – abnormalities of heart rhythm |  | I49.9 |
| **Heart failure** | |  |  |
|  | Heart failure, unspecified |  | I50.9 |
| **Cerebrovascular diseases** | |  |  |
|  | Non-traumatic intracranial haemorrhage |  | I62 |
|  | Cerebral infarction |  | I63 |
|  | Occlusion and stenosis of precerebral arteries without cerebral infarction |  | I65 |
|  | Occlusion and stenosis of cerebral arteries without cerebral infarction |  | I66 |
|  | Other cerebrovascular diseases |  | I67 |
|  | Cerebrovascular disorders in diseases classified elsewhere |  | I68 |
|  | Sequelae of cerebrovascular disease |  | I69 |
| **Atherosclerosis of native arteries of the extremities** | |  |  |
|  | Unspecified atherosclerosis of native arteries of the extremities |  | I70.20 |
|  | Atherosclerosis of native arteries of the extremities with intermittent claudication |  | I70.21 |
|  | Atherosclerosis of native arteries of the extremities with rest pain |  | I70.22 |
|  | Atherosclerosis of native arteries of the right leg with ulceration |  | I70.23 |
|  | Atherosclerosis of native arteries of the left leg with ulceration |  | I70.24 |
|  | Atherosclerosis of native arteries of the extremities with gangrene |  | I70.26 |
|  | Other atherosclerosis of native arteries of extremities |  | I70.29 |
| **Peripheral vascular disease** | |  |  |
|  | Other specified peripheral vascular disease |  | I73.8 |
|  | Peripheral vascular disease, unspecified |  | I73.9 |
| **Arterial embolism and thrombosis** | |  |  |
|  | Thromboembolic disease (embolism and thrombosis of unspecified artery) |  | I74.9 |
