# Supplementary material for: Cost of cardiovascular disease events in patients with and without type 2 diabetes and factors influencing cost: a retrospective cohort study
Source: BMC Public Health. 2024 Jul 26;24:2003. doi: 10.1186/s12889-024-19475-w (PMC11282681; doi:10.1186/s12889-024-19475-w)
Supplement: Supplementary file 2 — Supplementary Material 2. [file 12889_2024_19475_MOESM2_ESM.docx]

**Appendix B. ICD-10 codes used to identify cases of diabetes**

| **Definition** | | | | **ICD-10 codes** | |
| --- | --- | --- | --- | --- | --- |
| Type 2 diabetes mellitus | | | |  | E11 |
|  | Type 2 diabetes mellitus with hyperosmolarity | | |  | E11.0 |
|  |  | Without nonketotic hyperglycaemic-hyperosmolar coma (NKHHC) | |  | E11.00 |
|  |  | With coma | |  | E11.01 |
|  | Type 2 diabetes mellitus with ketoacidosis | | |  | E11.1 |
|  |  | Without coma | |  | E11.10 |
|  |  | With coma | |  | E11.11 |
|  | Type 2 diabetes mellitus with kidney complications | | |  | E11.2 |
|  |  | Type 2 diabetes mellitus with diabetic nephropathy | |  | E11.21 |
|  |  | Type 2 diabetes mellitus with chronic kidney disease | |  | E11.22 |
|  |  | Type 2 diabetes mellitus with other diabetic kidney complication | |  | E11.29 |
|  | Type 2 diabetes mellitus with ophthalmic complications | | |  | E11.3 |
|  |  | Type 2 diabetes mellitus with unspecified diabetic retinopathy | |  | E11.31 |
|  |  |  | With macular oedema |  | E11.311 |
|  |  |  | Without macular oedema |  | E11.319 |
|  |  | Type 2 diabetes mellitus with mild non-proliferative diabetic retinopathy | |  | E11.32 |
|  |  | Type 2 diabetes mellitus with moderate non-proliferative diabetic retinopathy | |  | E11.33 |
|  |  | Type 2 diabetes mellitus with severe non-proliferative diabetic retinopathy | |  | E11.34 |
|  |  | Type 2 diabetes mellitus with proliferative diabetic retinopathy | |  | E11.35 |
|  |  | Type 2 diabetes mellitus with proliferative diabetic cataract | |  | E11.36 |
|  |  | Type 2 diabetes mellitus with diabetic macular oedema, resolved following treatment | |  | E11.37 |
|  |  | Type 2 diabetes mellitus with other diabetic ophthalmic complication | |  | E11.39 |
|  | Type 2 diabetes mellitus with neurological complications | | |  | E11.4 |
|  |  | Type 2 diabetes mellitus with diabetic neuropathy, unspecified | |  | E11.40 |
|  |  | Type 2 diabetes mellitus with diabetic mononeuropathy | |  | E11.41 |
|  |  | Type 2 diabetes mellitus with diabetic polyneuropathy | |  | E11.42 |
|  |  | Type 2 diabetes mellitus with diabetic autonomic (poly)neuropathy | |  | E11.43 |
|  |  | Type 2 diabetes mellitus with diabetic amyotrophy | |  | E11.44 |
|  |  | Type 2 diabetes mellitus with other neurological complication | |  | E11.49 |
|  | Type 2 diabetes mellitus with circulatory complications | | |  | E11.5 |
|  |  | Type 2 diabetes mellitus with diabetic peripheral angiopathy without gangrene | |  | E11.51 |
|  |  | Type 2 diabetes mellitus with diabetic peripheral angiopathy with gangrene | |  | E11.52 |
|  |  | Type 2 diabetes mellitus with other circulatory complications | |  | E11.59 |
|  | Type 2 diabetes mellitus with other specified complications | | |  | E11.6 |
|  |  | Type 2 diabetes mellitus with diabetic arthropathy | |  | E11.61 |
|  |  |  | Type 2 diabetes mellitus with diabetic neuropathic arthropathy |  | E11.610 |
|  |  |  | Type 2 diabetes mellitus with other diabetic arthropathy |  | E11.618 |
|  |  | Type 2 diabetes mellitus with skin complications | |  | E11.62 |
|  |  | Type 2 diabetes mellitus with oral complications | |  | E11.63 |
|  |  | Type 2 diabetes mellitus with hypoglycaemia | |  | E11.64 |
|  |  | Type 2 diabetes mellitus with hyperglycaemia | |  | E11.65 |
|  |  | Type 2 diabetes mellitus with other specified complication | |  | E11.69 |
|  | Type 2 diabetes mellitus with unspecified complications | | |  | E11.8 |
|  | Type 2 diabetes mellitus without complications | | |  | E11.9 |
| Diabetic peripheral angiopathy | | | |  |  |
|  | Diabetes mellitus due to underlying condition with diabetic peripheral angiopathy | | |  | E08.5- |
|  | Drug- or chemical-induced diabetes mellitus with diabetic peripheral angiopathy | | |  | E09.5- |
|  | Type 2 diabetes mellitus with diabetic peripheral angiopathy | | |  | E11.5- |
|  | Other specified diabetes mellitus with diabetic peripheral angiopathy | | |  | E13.5- |
