# Supplementary material for: Cost of cardiovascular disease events in patients with and without type 2 diabetes and factors influencing cost: a retrospective cohort study
Source: BMC Public Health. 2024 Jul 26;24:2003. doi: 10.1186/s12889-024-19475-w (PMC11282681; doi:10.1186/s12889-024-19475-w)
Supplement: Supplementary file 3 — Supplementary Material 3. [file 12889_2024_19475_MOESM3_ESM.docx]

**Appendix C. ICD-10 codes used to designate cardiovascular risk**

| **Definition** | **ICD-10 codes** |
| --- | --- |
| Hypertension | I10, I16.0, I16.1, I11.9, I11.0, I12.9, I12.0, I13.10, I13.0, I13.11, I13.2, I15.0, I15.8, I15.1, N26.2, I15.2, I15.9 |
| Hyperlipidaemia | E780.x, E782x E784, E785 |
| Ischaemic heart disease | I20.0, I20.1x, I20.8x, I20.9x, I21.01, I21.02, I21.09, I21.19, I21.2x, I21.3x, I21.4x, I22.0, I22.1x, I22.2x, I22.8x, I22.9x, I24.0x, I24.1, I24.8x, I24.9x, I25.1x, I25.2x, I25.3x, I25.6x, I25.7x, I25.8x, I25.9x |
| Peripheral vascular disease | I70.20, I70.21, I70.22, I70.23, I70.24, I70.26, I70.29, I73.8, I73.9, I74.9 |
| Any stroke | I63.5x, I63.6x, I63.8x, I63.9x, I65.0x, I65.1x, I65.2x, I66.0x, I66.1x, I66.2x, I66.3x, I66.8x, I66.9x |
