# Supplementary material for: Cost of cardiovascular disease events in patients with and without type 2 diabetes and factors influencing cost: a retrospective cohort study
Source: BMC Public Health. 2024 Jul 26;24:2003. doi: 10.1186/s12889-024-19475-w (PMC11282681; doi:10.1186/s12889-024-19475-w)
Supplement: Supplementary file 4 — Supplementary Material 4. [file 12889_2024_19475_MOESM4_ESM.docx]

**Appendix D. DRG codes associated with ICD-10 diagnosis codes of interest**

| **ICD-10 code** | **ICD details** | **DRG code** |
| --- | --- | --- |
| I20‒I23 | Acute myocardial infarction | 05531, 05532, 05041, 05042, 05043, 05533, 05611, 05593, 10501, 10503, 12522, 18543, 01661, 03502, 04011, 05001, 05063, 05092, 05621, 06522, 08503, 09562 |
| I24, I25 | Ischaemic heart disease | 05532, 05533, 05003, 05612, 10501, 18542, 18543, 02532, 04643, 05001, 05002, 05003, 05092, 05093, 05101, 06573, 08501, 09562 |
| I11.0‒I12.0 | Hypertensive heart disease | 01513, 02531, 02532, 02533, 03501, 03502, 04511, 04502, 04571, 04573, 04583, 05512, 05513, 05611, 05612, 05613, 05571, 05572, 05573, 05511, 05512, 05513, 05593, 05631, 06533, 06563, 06572, 07522, 07531, 08501, 08502, 08503, 09551, 09561, 09562, 09563, 10511, 10501, 10502, 10503, 11501, 11502, 11503, 11581, 11582, 11583, 12522, 13501, 13553, 18541, 18542, 18543 |
| I62‒I69 | Stroke | 05571, 05572, 05573, 10502, 10503, 05611, 05612, 02531, 02532, 11581, 11582, 11583, 10512, 12521, 01503, 04502, 04503, 04583, 05043, 05593, 05613, 06143, 06571, 08501, 08503, 09551, 09561, 09562 |
| I50.9 | Heart Failure | 05573, 10502, 10503, 10512, 11581, 11582, 11583, 12521, 01503, 02531, 02532, 04502, 04503, 04583, 05043, 05571, 05572, 05573, 05593, 05611, 05612, 05613, 06143, 06571, 08501, 08503, 09551, 09561, 09562 |
| I42‒I46 | Cardiomyopathy and conduction disorder | 05541, 05542, 05543, 05561, 05562, 05063, 05593, 05611, 05612, 05613, 02532, 10501, 01502, 05611, 05563, 18542, 18543, 04013, 04503, 04543, 05021, 05041, 05102, 08501, 08503, 09562 |
| I70‒I73 | Atherosclerosis of peripheries and peripheral vascular disease | 05611, 05612, 10501, 04503, 05081, 05312, 05593, 05613, 08071, 08103, 08501, 08572, 08602 |
